# Phenylbutyrate increases activity of pyruvate dehydrogenase complex

**DOI:** 10.18632/oncotarget.1000

**Published:** 2013-04-28

**Authors:** Rosa Ferriero, Nicola Brunetti-Pierri

**Affiliations:** Telethon Institute of Genetics and Medicine, Naples, Italy; Telethon Institute of Genetics and Medicine, Naples, Italy and Department of Translational Medicine, Federico II University of Naples, Italy

Altered metabolism is an important hallmark of cancer cells and confers them a selective advantage for survival and proliferation. Metabolism in cancer cells is shifted towards glycolysis and decreased dependence on mitochondrial glucose oxidation, a phenomenon known as ‘Warburg effect' (aerobic glycolysis). Lactate, the end product of glycolysis, is produced in large excess by cancer cells that used it as metabolic fuel, and has been directly involved in cancer progression. Growth of cancer cells often occurs in a hypoxic microenvironment and thus, these cells rely on anaerobic glycolysis as a primary energy source. Nevertheless, conversion of glucose to lactate persists in cancer cells despite the presence of oxygen (aerobic glycolysis). This adaptation is initiated, in part, by activation of hypoxia-inducible factor 1α (HIF-1) that regulates expression of several glycolytic enzymes, glucose transporters, and mitochondrial enzymes, including pyruvate dehydrogenase kinases (PDKs) which inactivate the pyruvate dehydrogenase complex (PDHC) [1]. The PDHC is an important enzyme in metabolism linking glycolysis to the tricarboxylic acid (TCA) cycle and lipogenic pathway. PDHC catalyzes in mitochondria the irreversible conversion of pyruvate into acetyl-CoA that initiates the TCA cycle. PDHC is formed by three different enzymes: thiamine diphosphate-dependent heterotetrameric (α_2_β_2_) E1, dihydrolipoamide acetyltransferase (E2), and FAD containing dihydrolipoamide dehydrogenase (E3), that is integrated into the complex by an E3-binding protein (E3BP). Phosphorylation of specific E1α serine residues by PDKs (PDK1, PDK2, PDK3, and PDK4) results in inactivation of PDHC, whereas dephosphorylation by pyruvate dehydrogenase phosphatases (PDP1 and PDP2) restores PDHC activity (Fig. 1). Inhibition of PDHC activity results in conversion of pyruvate into lactate by lactate dehydrogenase (LDH) in the cytoplasm.

**Figure 1:**
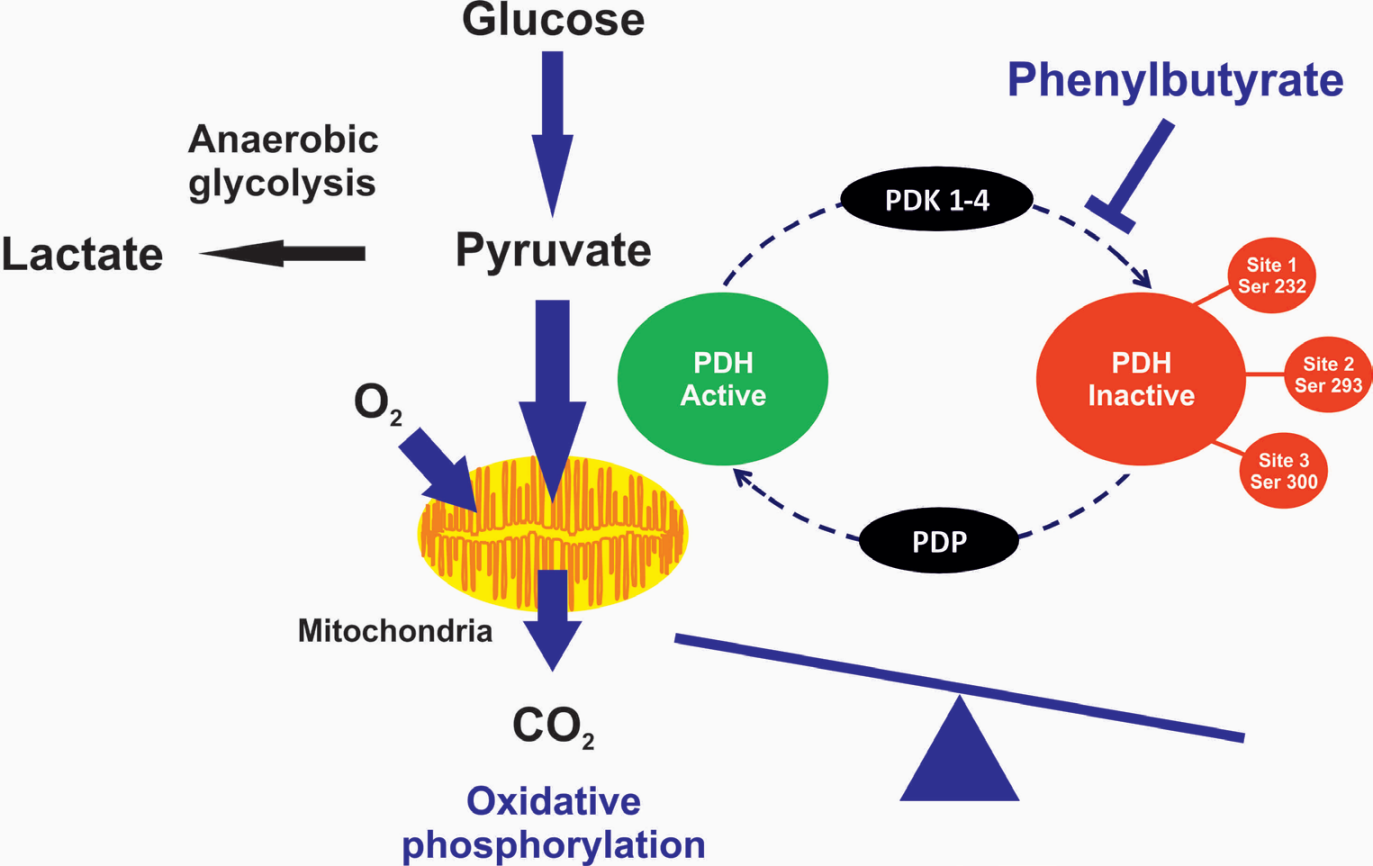
Regulation of pyruvate dehydrogenase complex (PDHC) activity and its role in pyruvate metabolism and oxidative phosphorylation The phosphorylation is catalyzed by pyruvate dehydrogenase kinases (PDKs) whereas the dephosphorylation is catalyzed by phosphatases (PDPs). Phenylbutyrate-mediated inhibition of PDK results in increased proportion of activated PDHC. The greater PDHC activity enhances glucose oxidation, acetyl-coA levels, tricarboxylic acid cycle, and electron transport chain activity.

Deficiency of PDHC is one of the most common inborn errors of energy metabolism and affected patients present with progressive neurological degeneration and lactic acidosis [2]. Dichloroacetate (DCA) increases PDHC activity by inhibition of PDK and is effective in reducing blood and tissue lactate [3]. However, DCA has been associated with hepatocellular and peripheral nerve toxicity. We have recently found that phenylbutyrate, a drug used in patients with urea cycle defects and cancer, results in reduction of phosphorylated E1α and increased enzyme activity both in fibroblasts and mice (Fig. 1). Moreover, it improved the morphological, locomotor, and biochemical abnormalities of a zebrafish model of PDHC deficiency and it prevented systemic lactic acidosis induced by partial hepatectomy in mice [2]. The effect of phenylbutyrate was not dependent on increased expression of genes encoding PDHC subunits or its regulatory enzymes (PDKs and PDPs) and thus, it is not secondary to its known activity as histone deacetylase (HDAC) inhibitor [4]. Phenylbutyrate is a drug already approved for use in humans with a well-established safety profile, and therefore the study by Ferriero et al. has the potential to be rapidly translated into a treatment for patients with PDHC deficiency and lactic acidosis [2].

Metabolic remodeling in cancer cells is clearly involved in cancer progression and can be exploited for cancer treatment. Through enhancement of PDHC activity, DCA promotes mitochondrial glucose oxidation and thus reverses the cancer metabolic remodeling. Based on this effect, DCA has been investigated as an anti-cancer agent. The clinical evidence that DCA, as a single agent, might be effective in glioblastoma [5] suggests its use in other glycolytic forms of cancer. Interestingly, phenylbutyrate also has shown efficacy in different cancers and so far, its anti-tumor activity has been attributed to the HDAC inhibitor mode of action [4]. Based on our recent finding [2], the therapeutic effect of phenylbutyrate on cancer cells may depend upon increased PDHC activity (Fig. 1), in addition or alternatively to its proposed role as HDAC inhibitor. Phenylbutyrate might be a safer alternative to DCA especially for patients treated with chemotherapies such as taxane, platinum and vinca-alkaloid that like DCA can also result in peripheral neuropathy [3].

Metabolic tissues in mammals transform ingested food into glucose, amino acid, and lipids to meet the metabolic needs of both differentiated and proliferating cells. Alterations in the appropriate balance of fuels and/or signal transduction pathways that deal with nutrient utilization may underlie cancer predisposition associated with metabolic diseases such as diabetes and obesity. Excess calories are indeed associated with shortened life span whereas caloric deprivation prolongs life span. Intriguingly, both phenylbutyrate and DCA were found to increase life span in *Drosophila melanogaster* and *Caenorhabditis elegans*, respectively [6-7]. Treatment of obese and diabetic mice with phenylbutyrate resulted in normalization of hyperglycemia, restoration of systemic insulin sensitivity, resolution of fatty liver disease, and enhancement of insulin action [8]. It has been proposed that this effect is dependent on phenylbutyrate acting as a chemical chaperone, another mode of action of phenylbutyrate. However, given the role of PDHC in energy metabolism, and the increased glucose disposal in peripheral tissues obtained by increased PDHC activity, the effect on glucose homeostasis may be also related to the stimulatory effect of phenylbutyrate of PDHC.

In summary, the multiple biological effects observed in normal and cancer cells treated with phenylbutyrate can be dependent, at least in part, on the newly recognized activity on PDHC, a key enzyme for metabolism and survival.

## References

[1] Kim JW (2006). Cell Metab.

[2] Ferriero R (2013). Sci Transl Med.

[3] Stacpoole PW (2008). Pediatrics.

[4] Bolden JE (2006). Nat Rev Drug Discov.

[5] Michelakis ED (2010). Sci Transl Med.

[6] Kang HL (2002). Proc Natl Acad Sci U S A.

[7] Schaffer S (2011). Biogerontology.

[8] Ozcan U (2006). Science.

